# Validation of the psychosocial impact of dental aesthetics questionnaire (Pidaq) in Spanish adolescents

**DOI:** 10.4317/medoral.18324

**Published:** 2012-12-10

**Authors:** José M. Montiel-Company, Carlos Bellot-Arcís, José M. Almerich-Silla

**Affiliations:** 1 Post-Doctoral Assistant Professor, Department of Stomatology, University of Valencia, (Spain); 2 Orthodontist, Department of Stomatology, University of Valencia, (Spain); 3 Tenured Lecturer, Department of Stomatology, University of Valencia, (Spain)

## Abstract

Objectives: The purpose of this study was to assess the validity and reliability of the Spanish version of PIDAQ for application in adolescents. Study Design: The questionnaire was translated, cross-culturally adapted and completed by 627 adolescents (366 12-year-olds and 261 15-year-olds). The adolescents were also examined by 4 examiners who had been calibrated against a gold standard and relative to each other (Kappa >0.85) in determining treatment need with the Dental Aesthetic Index (DAI) and the Index of Orthodontic Treatment Need (IOTN) DHC and AC components. Results: Cronbach´s alpha of the translated PIDAQ was 0.90. The 23 items of the questionnaire were divided into four domains that explained 60% of the variance. The test-retest reliability of the questionnaire was 0.93. Discriminant validity revealed a significant association between the scores for the questionnaire and its subscales or domains and those for the DAI, IOTN-DHC and IOTN-AC treatment need indices. Adolescents with orthodontic treatment need scored higher in the questionnaires. Conclusions: The results show that the Spanish version of PIDAQ has a very similar internal structure and psychometric properties to those of the original questionnaire and demonstrate its validity for use with Spanish adolescents.

** Key words:**Orthodontics, epidemiology, quality of life, malocclusion.

## Introduction

Epidemiological studies of malocclusion prevalence have shown that this condition affects many people worldwide. Malocclusion affects function and aesthetics, but it also has important social, psychological and financial repercussions (1). Numerous occlusal or orthodontic treatment need indices have attempted to analyze the anatomical and aesthetic aspects of malocclusion (2), but ignored the patients’ perceptions of their own malocclusions or how these influence their welfare or their quality of life (3). The differences between the professionals’ and the patients’ perceptions of aesthetic effect and orthodontic treatment need are considerable (4), and the psychosocial consequences that may arise from a particular malocclusion cannot be ignored.

Health-related quality of life (HRQoL) measurement tools provide information on the patient’s perception of his or her welfare in relation to a particular medical condition (5).

A number of studies have shown the negative impact that oral disorders can have on both the patients and their families (6-8). As a result, greater interest is being shown in the use of questionnaires that offer more information on the patients’ quality of life in relation to their oral health (3,9,10), and to their perception of their own appearance (10,11).

The Psychosocial Impact of Dental Aesthetics Questionnaire (PIDAQ) is a tool which gives very valuable information on aspects of the oral health-related quality of life (OHRQoL). This self rating instrument was designed to assess the psychosocial impact of dental aesthetics in young adults (5).

Most questionnaires, including the PIDAQ, were developed in English-speaking countries and written in English. When they are used in other countries they need to be translated and adapted appropriately, taking into account the cultural and social aspects of the new region where they are to be used while preserving their psychometric properties (12).

Brazilian and Chinese versions of the PIDAQ have been published recently (12,13), but no Spanish version has yet been published in any international journal. Prompted by the importance that this type of questionnaire has acquired and the number of people worldwide that have Spanish as their first language, this study aimed to adapt the PIDAQ for a Spanish-speaking public and assess its validity.

## Patient and Methods

-Description of the PIDAQ

The PIDAQ is a psychometric instrument containing 23 items. Structurally, it is composed of four subscales, one positive and three negative, which represent 4 domains: aesthetic concern (AC; 3 items), psychological impact (PI; 6 items), social impact (SI; 8 items) and dental self-confidence (DSC; 6 items). A five-point Likert scale is used, ranging from 0 (no impact of dental aesthetics on QoL) to 4 (maximal impact of dental aesthetics) for each item. The response options are as follows: 0=not at all; 1=a little; 2=somewhat; 3=strongly; and 4=very strongly (5).

-Translation and cross-cultural adaptation of the PIDAQ

The PIDAQ was first translated into Spanish by two separate translators who then worked together to produce the initial draft. Two different translators separately back-translated this draft into English. A committee made up of two orthodontists and two dentists with QoL and oral health assessment expertise and fluency in English assessed the semantic and conceptual equivalence of the 23 items and adapted them for the Spanish version of the PIDAQ.

-Pilot study

The Spanish version was pilot tested on a convenience sample of 30 adolescents attending a secondary school in the city of Valencia (Spain). The pilot test demonstrated that the Spanish version of the PIDAQ exhibited appropriate semantic and conceptual equivalence.

-Assessment of validity and reliability of the Spanisversion of the PIDAQ

The validity and reliability assessment of the Spanish version of the PIDAQ was carried out during the November-December 2010 epidemiological study of oral health among schoolchildren in the Valencia region of Spain, which covered a sample population of 42 schools selected at random from the region’s total of 1200 schools.

Adolescents whose anterior teeth presented visible caries lesions, traumas, dental hypoplasias or fluorotic lesions or who were undergoing orthodontic treatment were excluded from the analysis. The final sample included the questionnaires of 627 pupils: 366 12-year-olds and 261 15-year-olds. A month later, a random sample of 32 adolescents repeated the questionnaire for the purposes of determining test-retest reliability.

-Determination of orthodontic treatment need 

The Dental Aesthetic index (DAI) and the Dental Health Component (IOTN-DHC) and Aesthetic Component (IOTN-AC) of the Index of Orthodontic Treatment Need were used to assess the adolescents’ orthodontic treatment need. The examinations were performed in the schools by 4 examiners who had been calibrated against a gold standard and relative to each other (Kappa >0.85) in measuring these three indices.

The sample was divided into the 4 DAI grades: scores of 25 or less show normal or minor malocclusion, scores of 26-30 definite malocclusions with elective treatment; scores of 31-35 severe malocclusions with treatment highly desirable; and scores of 36 and higher very severe or disabling malocclusions with treatment considered mandatory.

The sample was also divided into 3 IOTN-DHC groups (grades 1-2, grade 3 and grades 4-5) and 3 IOTN-AC groups (score 1-4, score 5-7, and score 8-10).

-Statistical analysis

The Statistical Package for the Social Sciences v. 18.0® was used for data analysis. Descriptive analyses were performed (mean and standard deviation of the four subscales and of the PIDAQ questionnaire as a whole). To study the questionnaire's psychometric properties and calculate the total score, the variables in the dental self confidence subscale (items 1 to 6) were re-coded to bring the direction of the scores into line with the other 3 subscales. A factor analysis of the questionnaire was carried out and internal consistency was measured by Cronbach´s alpha.

Test-retest reliability was assessed by calculating the intraclass correlation coefficient. Discriminant validity was tested by comparing the groups classified according to their DAI, IOTN-DHC and IOTN-AC scores with the scores for each subscale and for the PIDAQ total. ANOVA was employed to evaluate the differences between means.

-Ethical considerations

The study was approved by the Ethical Committee of the University of Valencia and the recommendations for this type of study were followed. The parents' informed consent was requested before conducting the examinations and administering the questionnaire.

## Results

-Reliability

The internal consistency of the questionnaire assessed by Cronbach´s alpha coefficient was 0.901; the standardized Cronbach´s alpha was 0.904. The item and scale correlation coefficients were between 0.39 and 0.69. The reliability of the 4 subscales was 0.900 for dental self-confidence, 0.862 for social impact, 0.808 for psychological impact and 0.768 for aesthetic concern. The correlation coefficients of item and subscales were >0.4.

-Construct validity

The Kaiser-Meyer-Olkin measure of sampling adequacy was 0.920 and the Bartlett´s test of sphericity was 7048.9 (p=0.00). Principal com-ponents analysis extracted the same four dimensions as the original questionnaire ( Table 1).

**Table 1:**
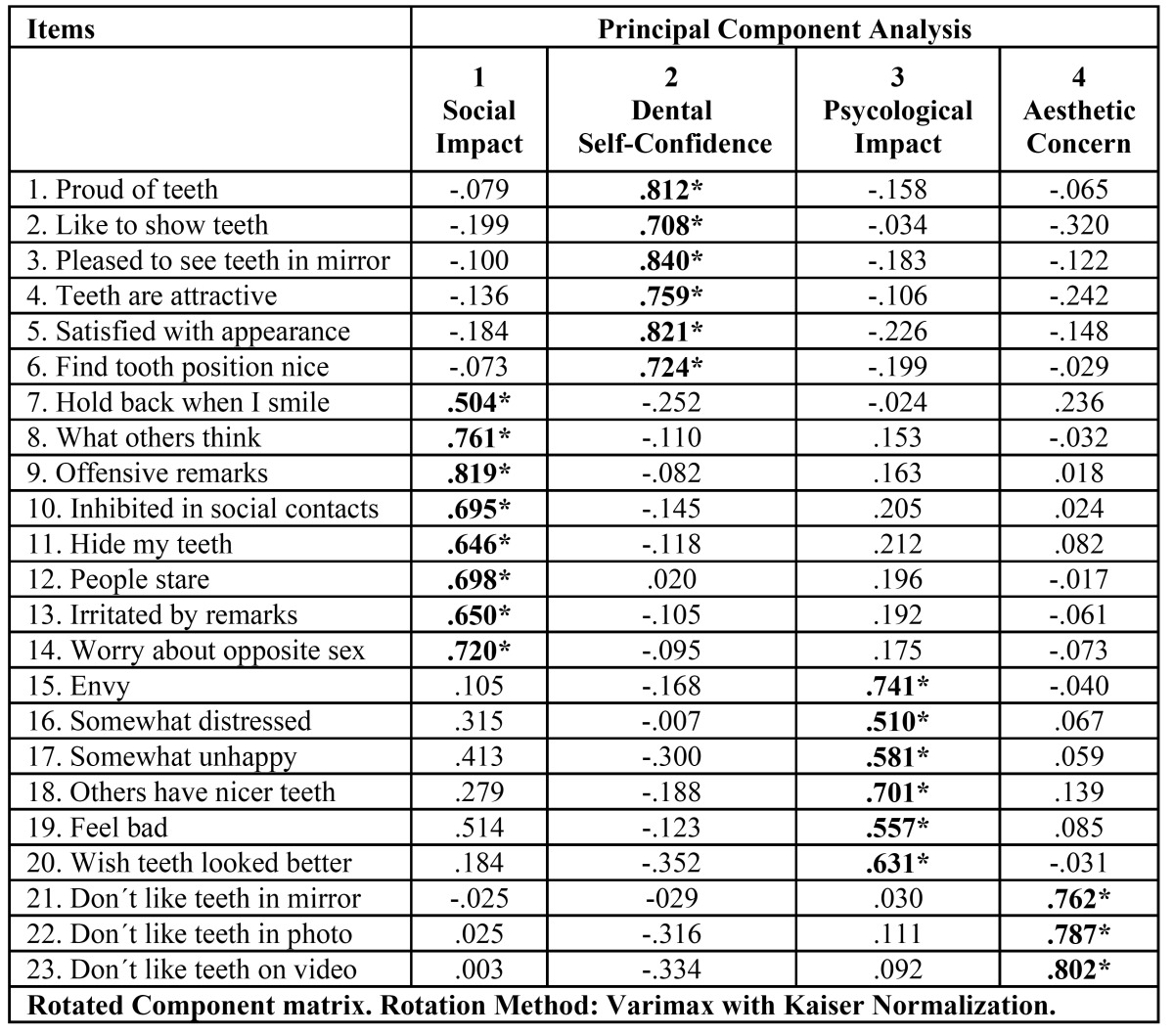
Factor loadings of the items of the psychological impact of dental aesthetics questionnaire (PIDAQ) subscales after principal component analysis.

Common factor 1 contained the original Social Impact subscale items 7-14 (eigenvalue=7.70) and explained 33.48 % of the variance. Common factor 2 contained items 1-6, comprising the Dental Self Confidence subscale (eigenvalue=3.4), and explained 14.65% of the variance. Common factor 3 contained the same items 15-20 as the Psychological Impact subscale (eigenvalue=1.52) and explained 6.61% of the variance. Finally, common factor 4 contained the items 21-23 of the Aesthetic Concern subscale (eigenvalue=1.21) and explained 5.28% of the variance. In total, these 4 components explained 60.03% of the total variance.

-Reproducibility

The test-retest reliability of the PIDAQ was determined: the intraclass correlation coefficient was 0.93 for the PIDAQ and ranged between 0.87 and 0.93 for the 4 subscales.

-Discriminant validity

Significant differences in the median scores for the Dental Self-Confidence (DSC), Social Impact (SI) and Psychological Impact (PI) subscales and the total PIDAQ scale were found between DAI score groups ( Table 2). The median scores for PIDAQ and all its subscales differed significantly between the groups classified by IOTN-DHC grades ( Table 3), while significant differences between the median scores of the groups categorized according to IOTN-AC criteria were found in PIDAQ and all subscales except Aesthetic Concern ( Table 4).

**Table 2:**
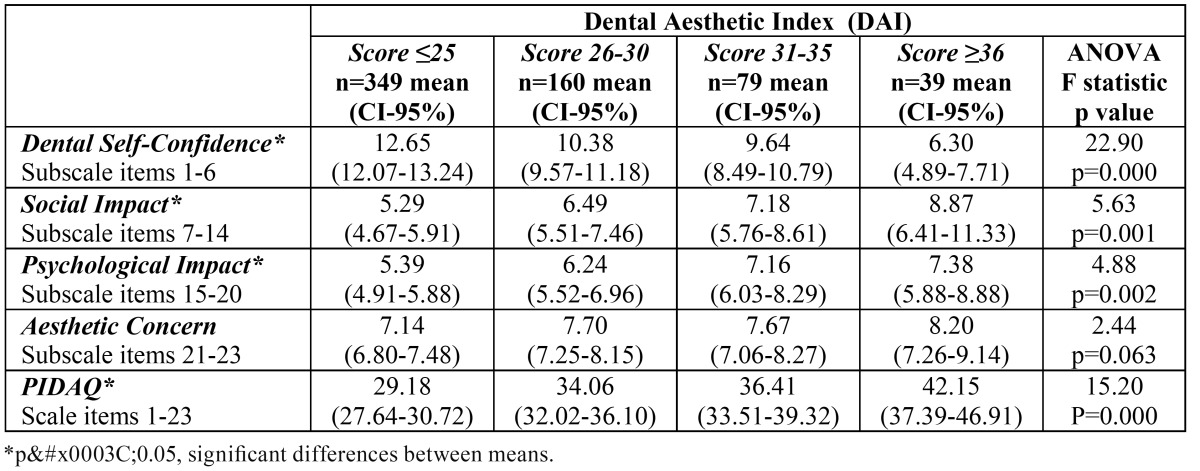
Subscales and PIDAQ scores according to Dental Aesthetic Index categorization.

**Table 3:**
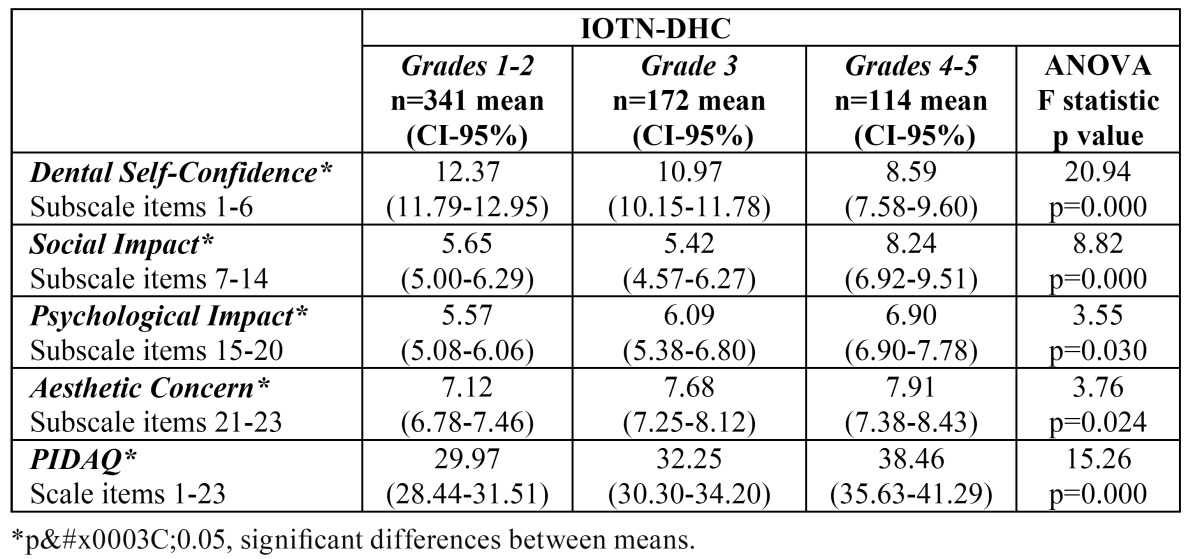
Subscales and PIDAQ scores according to Index of Orthodontic Treatment Need - Dental Health Component (IOTN-DHC) categorization.

**Table 4:**
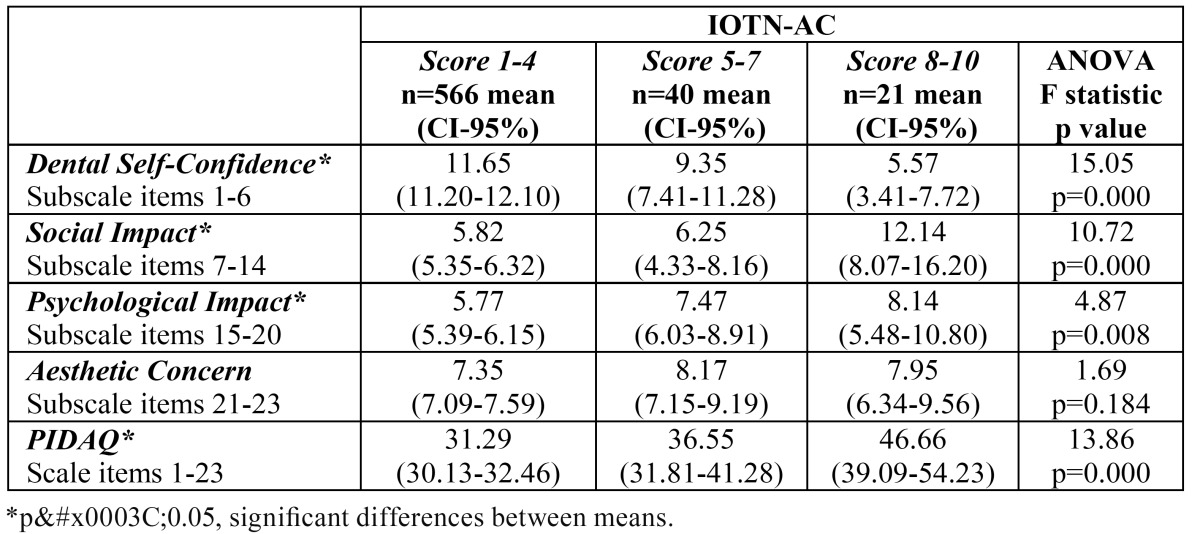
Subscales and PIDAQ scores according to Index of Orthodontic Treatment Need - Aesthetic Component (IOTN-AC) categorization.

## Discussion

Several questionnaires have been developed recently to analyze the patients’ perception of their malocclusions and their oral health related quality of life (OHRQoL). When we want to use a particular questionnaire in another country, we not only have to translate it but also need to adapt it to the different social and cultural circumstances. This questionnaire can then be validated for use in other regions, ensuring that the results will be comparable with those of other studies. This will only be possible if the questionnaire shows good psychome-tric properties.

Factor analysis has shown that the structure of our Spanish PIDAQ questionnaire is the same as that developed by Klages and cols. (5) and employed by Sardenberg et al. (12), in the Brazilian version of PIDAQ. It has four domains, represented by the Aesthetic Concern (AC), Psychological Impact (PI), Social Impact (SI) and Dental Self-Confidence (DSC) subscales, which together explain 60% of the variance. The Chinese version joined PI and AC into a single subscale, essentially because of the cultural characteristics of the young Chinese population, as the authors explained (13).

The present study was carried out in a representative sample numbering 627 children from 12 to 15 years of age. Members of this sector of the population are particularly concerned with their image, which plays an important part in both their psychological welfare and their social success (14,15). This could explain why the AC subscale score was higher than in other studies (12).

The Spanish version of the PIDAQ has shown good reproducibility, as Cronbach’s alpha was 0.93 for the PIDAQ as a whole and between 0.87 and 0.93 for the 4 subscales. Reproducibilities above 0.74 can be considered excellent. On comparing these figures with the original study conducted by Klages et al. (5) and with those obtained by Sardenberg et al. (12) and Lin et al. (13) in their respective studies, the Spanish version of the questionnaire obtained similar results.

The discriminant validity of the questionnaire and its 4 subscales has been shown by relating it to the treatment need determined by the DAI, IOTN-DHC and IOTN-AC indices. The overall PIDAQ and PI and SI subscale scores increased as the treatment need rose, displaying a significant positive relationship when measured by any of the three indices employed. The AC subscale was only related significantly to IOTN-DHC. The DSC subscale, on the other hand, showed a significant negative relationship. The reason is that the scores for this subscale were not re-coded to bring their direction into line with the other 3 subscales, as was done when calculating the total PIDAQ score.

As regards AC, no significant association between this subscale and the treatment need as measured by DAI was found by Sardenberg and cols. Either (12). One possible explanation is that the adolescent population has always been particularly concerned with appearance and aesthetics, sometimes not in a mature or objective way, and this could influence the results (16,17). On occasion, the patient’s perception of his or her malocclusion is not related to standard treatment need as determined objectively by the indices (16,18,19). Moreover, patients’ perceptions of malocclusion are usually quite different from those of the specialist (18,20). It should also be remembered that as in similar studies (12), most of the sample presented normal occlusion or only slight malocclusion, which is habitually the case when representative samples are employed.

The results show that the Spanish version of PIDAQ has a very similar internal structure and psychometric properties to those of the original questionnaire by Klages et al. (5), as well as excellent reproducibility, and can validly be used with Spanish adolescents.

## References

[1] Bernabé E, Tsakos G, De Oliveira CM, Sheiham A (2008). Impacts on daily performances attributed to malocclusions using the condition-specific feature of the Oral Impacts on Daily Performances Index. Angle Orthod.

[2] Onyeaso CO, Aderinokun GA (2003). The relationship between Dental Aesthetic Index (DAI) and perceptions of aesthetics, function and speech amongst secondary school children in Ibadan, Nigeria. Int J Paediatr Dent.

[3] de Oliveira CM, Sheiham A, Tsakos G, O'Brien KD (2008 ). Oral health-related quality of life and the IOTN index as predictors of children's perceived needs and acceptance for orthodontic treatment. Br Dent J.

[4] Klages U, Bruckner A, Zentner A (2004). Dental aesthetics, selfawareness, and oral health-related quality of life in young adults. Eur J Orthod.

[5] Klages U, Claus N, Wehrbein H, Zentner A (2006). Development of a questionnaire for assessment of the psychosocial impact of dental aesthetics in young adults. Eur J Orthod.

[6] Locker D, Jokovic A, Stephens M, Kenny D, Tompson B, Guyatt G (2002). Family impact of child oral and oro-facial conditions. Community Dent Oral Epidemiol.

[7] de Oliveira CM, Sheiham A (2004). Orthodontic treatment and its impact on oral health-related quality of life in Brazilian adolescents. Journal of Orthod.

[8] Marques LS, Ramos-Jorge ML, Paiva SM, Pordeus IA (2006). Malocclusion: esthetic impact and quality of life among Brazilian schoolchildren. Am J Orthod Dentofacial Orthop.

[9] Kok YV, Mageson P, Harradine NWT, Sprod AJ (2004). Comparing a quality of life measure and the Aesthetic Component of the Index of Orthodontic Treatment Need (IOTN) in assessing orthodontic treatment need and concern. J Orthod.

[10] Birkeland K, Bøe OE, Wisth PJ (2000). Relationship between occlusion and satisfaction with dental appearance in orthodontically treated and untreated groups. A longitudinal study. Eur J Orthod.

[11] Bos A, Hoogstraten J, Prahl-Anderson B (2003). Expectations of treatment and satisfaction with dentofacial appearance in orthodontic patients. Am J Orthod Dentofacial Orthop.

[12] Sardenberg F, Oliveira AC, Paiva SM, Auad SM, Vale MP (2011). Validity and reliability of the Brazilian version of the psychosocial impact of dental aesthetics questionnaire. Eur J Orthod.

[13] Lin H, Quan C, Guo C, Zhou C, Wang Y, Bao B Translation and validation of the Chinese version of the psychosocial impact of dental aesthetics questionnaire. Eur J Orthod.

[14] DiBiase AT, Sandler PJ (2001). Malocclusion, orthodontics and bullying. Dent Update.

[15] Onyeaso CO, Sanu OO (2005). Perception of personal dental appearance in Nigerian adolescents. Am J Orthod Dentofacial Orthop.

[16] Bernabé E, Flores-Mir C (2006). Normative and self-perceived orthodontic treatment need of a Peruvian university population. Head Face Med.

[17] Hassan AH, Amin-Hel S (2010). Association of orthodontic treatment needs and oral health-related quality of life in young adults. Am J Orthod Dentofacial Orthop.

[18] Tang EL, So LL (1995). Correlation of orthodontic treatment demand with treatment need assessed using two indices. Angle Orthod.

[19] Bellot-Arcís C, Montiel-Company JM, Manzanera-Pastor D, Almerich-Silla JM (2012 ). Orthodontic treatment need in a Spanish young adult population. Med Oral Patol Oral Cir Bucal.

[20] Soh J, Sandham A (2004). Orthodontic treatment need in Asian adult males. Angle Orthod.

